# Seeing the Invisible Resiliency (STIR): Chronic Autoimmune Conditions and Post‐Secondary Education Experiences in Young Adulthood

**DOI:** 10.1111/hex.70332

**Published:** 2025-07-11

**Authors:** Samantha A. Morin, Angelina Horta, Katelyn Greer, Parveen Priya Rai, Haley Gross, Raegan Reiter, Ingrid Nielssen, Marcia Bruce, Kim Giroux, Deborah A. Marshall

**Affiliations:** ^1^ Patient and Community Engagement Research (PaCER) University of Calgary Calgary Canada; ^2^ Child Health & Exercise Medicine Program McMaster University Hamilton Canada; ^3^ Faculty of Health Sciences McMaster University Hamilton Canada; ^4^ School of Interdisciplinary Science McMaster University Hamilton Canada; ^5^ Department of Food and Human Nutritional Sciences University of Manitoba Winnipeg Canada; ^6^ Professional Master's Dietetics Program SickKids Hospital and Toronto Metropolitan University Toronto Canada; ^7^ Alberta College of Social Workers (ACSW) Edmonton Canada; ^8^ Department of Community Health Sciences, Cumming School of Medicine University of Calgary Calgary Canada; ^9^ Department of Medicine, Cumming School of Medicine University of Calgary Calgary Canada; ^10^ Alberta Children's Hospital Research Institute (ACHRI) University of Calgary Calgary Canada

**Keywords:** accessibility, accommodations, chronic autoimmune condition, patient‐oriented, post‐secondary education, young adult

## Abstract

**Introduction:**

Young adults with chronic autoimmune conditions face unique and often overlooked challenges in post‐secondary education due to the invisible and unpredictable nature of these conditions. This patient‐led qualitative study aims to further understand the experiences of young adults living with chronic autoimmune conditions while attending or considering attending post‐secondary education.

**Methods:**

The study followed the three‐phase Patient and Community Engagement Research (PaCER) approach, a participatory framework that trains individuals with lived experience to lead all stages of research. In the first stage (SET), the protocol was co‐designed with three external patient‐partners. Study participants included young adults (18–35 years) with a chronic autoimmune condition for > 1 year who considered attending or attended a Canadian post‐secondary school within the last 5 years and were recruited through social media. Data were collected (COLLECT) via focus group and interviews and then analysed using thematic and narrative analysis. Findings were shared back with study participants (REFLECT) for refinement and to inform recommendations.

**Results:**

Ten young adults participated, and eight key themes were identified. Themes included the wide‐ranging impacts of disease management, the value of peer and family support, protective and risk factors for success, limited awareness and education around chronic conditions, and sometimes‐unconscious burden of navigating invisible conditions. Participants also reflected on their resilience and the shifting accessibility landscape during Covid‐19, and offered detailed feedback on current gaps and needed support. Their recommendations underscored ongoing institutional shortcomings and the need for systemic change.

**Conclusion:**

Our findings indicate that young adults living with chronic autoimmune conditions are not having their needs sufficiently met while navigating the post‐secondary education system. It is imperative that changes and feedback provided by students with lived experience are implemented to ensure an accessible post‐secondary education experience.

**Patient or Public Contribution:**

Seven PaCER researchers, who identify as young adults with lived experience of chronic conditions, led the study design, data collection, analysis and manuscript preparation. This study was also co‐designed with three external patient‐partners who also identify as young adults with chronic conditions.

## 1 Introduction

Students with chronic conditions face unique challenges in post‐secondary education, including any formal or informal education pursued after high school (secondary school). These challenges may significantly impact their academic performance, mental health and overall well‐being [1, 2]. Research indicates that the post‐secondary education system often lacks adequate understanding and resources to support these students, leading to barriers that prevent them from reaching their full potential or attending altogether, as observed in international contexts including Canada, the United States and the United Kingdom [1, 2, 3]. Our team of patient researchers aligned with this literature based on their lived experiences and established an initial research question ‘*How is the experience of navigating post‐secondary education in young adulthood (18–30 years) impacted by living with chronic conditions?*’ After discussions with our external patient‐partners and further review of the literature, we refined our research to focus on autoimmune conditions (see ‘Methods’ section for a full description of the participatory approach).

Chronic autoimmune diseases or conditions occur when the immune system mistakenly attacks the body's own tissues [4]. These conditions can affect various organs and systems, leading to a wide range of symptoms and complications [4, 5]. Some of the most common autoimmune conditions among young adults include type 1 diabetes, inflammatory bowel disease and inflammatory arthritis [5]. The prevalence of autoimmune conditions varies with each type, but overall, they impact about 1%–5% of the population worldwide, with rates continuing to rise [5, 6].

These conditions share a commonality as they present with unique challenges and experiences due to being ‘invisible’ in nature and the unpredictability of the presence and intensity of symptoms [4, 5], which was emphasised by our team and patient‐partners as significant barriers in post‐secondary settings. Individuals with other invisible conditions, like mental health disorders, have been overlooked as a group requiring accommodations in the context of post‐secondary education [2]. However, other conditions, such as dyslexia, have seen increasing recognition and support, likely due in part to greater public awareness and advances in assistive technology [7]. Despite the increasing prevalence of autoimmune conditions, there is a lack of comprehensive research examining the experiences of young adults with these conditions who attended or considered attending post‐secondary education. A better understanding of their challenges and needs can inform accessibility planning, policy development and systemic changes. This includes areas such as advocacy, access to effective supports and resources, and increasing awareness and understanding of chronic autoimmune conditions to reduce stigma. Therefore, this study aims to understand the experiences of young adults living with chronic autoimmune conditions in accessing supports and resources for attending or considering attending post‐secondary education in Canada.

## 2 Materials and Methods

### 2.1 Study Overview

This qualitative study followed the Patient and Community Engagement Research (PaCER) approach. The PaCER approach is a participatory qualitative methodology that centres patients in all phases of the research process (see Table 4: GRIPP2‐SF Checklist for Reporting Patient and Public Involvement). This study was conducted as part of the University of Calgary PaCER training programme, which aims to teach those with lived experience how to generate patient‐informed qualitative health research evidence that can improve patient‐centred health system planning, practice and policy using the PaCER approach [8, 9, 10]. This study was carried out by a group of seven PaCER‐trained researchers who have lived experience with chronic conditions and have attended or considered post‐secondary education.

**Figure 1:**
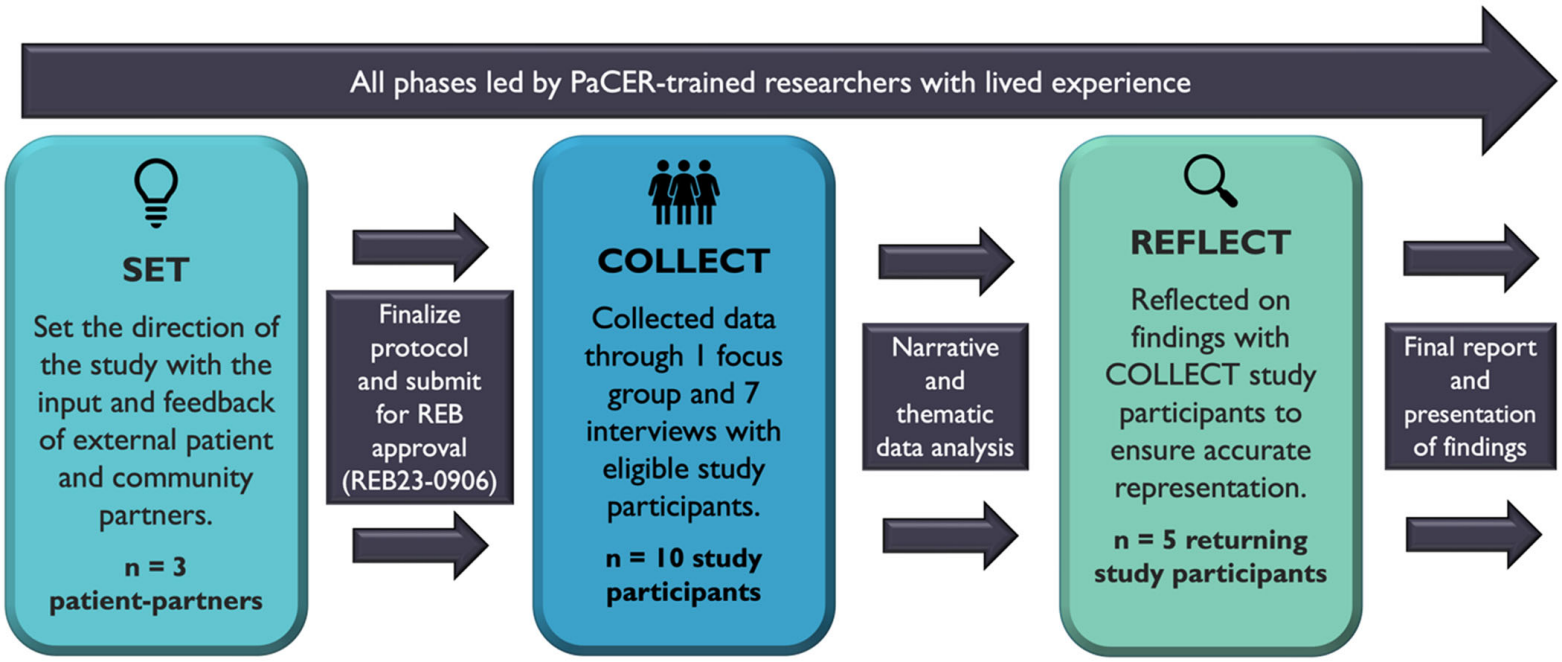
PaCER Research Methodology Framework. This study followed the PaCER approach, which follows three specific stages—SET, COLLECT and REFLECT. All stages of the research were led by the PaCER researchers. SET engaged three external patient‐partners, COLLECT engaged ten study participants and REFLECT engaged five of the COLLECT study participants.

The PaCER approach has three phases: SET, COLLECT and REFLECT (see Figure 1). The SET phase engaged patients‐partners, who were young adults with lived and living experience of chronic conditions (*n* = 3), in designing the scope and direction of the study. Data were then collected (COLLECT phase) by the PaCER researchers from eligible study participants (*n* = 10) using focus groups and interviews. These data were thematically analysed, and the results were shared back with study participants in a REFLECT focus group or interview. In the REFLECT phase, preliminary themes and recommendations were verified for accurate representation by study participants (from COLLECT phase) who agreed to participate (*n* = 5). Ethics approval was obtained from the University of Calgary Conjoint Health Research Ethics Board (REB) (REB23‐0906) before data collection.

### 2.2 Patient‐Partner and Study Participant Recruitment

The SET patient‐partners and COLLECT study participants were recruited through collaborations with the Alberta Children's Hospital Research Institute (ACHRI) and the Alberta SPOR SUPPORT Unit (AbSPORU) Patient Engagement Team. Recruitment efforts included the use of websites, newsletters, email distribution to organisational mail lists, and social media platforms (Twitter, Facebook and Instagram) of these organisations and the PaCER researchers. For study participants, a participant interest form was created to confirm eligibility and preference for a focus group or individual interview. To ensure eligibility, participants were asked to confirm their selected age range when contacted. Those who confirmed the same age range were considered eligible and were followed up to schedule a focus group or interview.

### 2.3 Co‐Design

#### 2.3.1 SET Phase

As briefly discussed in the ‘Introduction’, the patient researchers (now known as PaCER researchers) developed the initial research topic based primarily on their personal lived experiences. A targeted review of existing literature was also conducted to support the relevance of the topic and confirm it was underexplored. Three external patient‐partners were recruited to join a Zoom discussion group to finalise the research question (see Appendix 1 for the SET discussion group guide). Eligibility criteria for patient‐partners included being aged 18–30 years, Canadian or Permanent Residents, living with a chronic condition for at least one year, and having considered or attended a Canadian post‐secondary school within the last five years. Patient‐partners helped narrow the study focus to chronic autoimmune conditions, expand the age range to include up to age 35, and refine the language for clarity and inclusivity.

### 2.4 Data Collection and Analysis

#### 2.4.1 COLLECT Phase

Once the protocol was finalised and ethics approval was attained, all 10 eligible patients who responded to our interest form were recruited to participate in COLLECT focus groups or interviews in October and November 2023. Individuals who met the following inclusion criteria were eligible to participate in the study:

- Between the ages of 18–35 years (as patient‐partners indicated that young adults with chronic conditions may experience delayed or interrupted education due to illness).
- Considered or attended any form of post‐secondary education or schooling—includes any public and for‐profit colleges, universities and institutions as well as certificate programmes, diploma programmes, bachelor's degrees, trade school and so forth within the last five years.
- Living with (self‐identified) at least one chronic autoimmune condition or autoimmune‐like condition (e.g., endometriosis) for over one year (to ensure patients have some level of awareness and understanding of their own condition and needs) at the time of consideration/attendance of post‐secondary education.
- Considered or would be considered a domestic student by Canadian post‐secondary schools (i.e., Canadian Citizen or Permanent Resident), as international students have access to different resources and supports, which require a focused approach to understand each experience.

Both focus groups and interviews were offered to provide participants flexibility with scheduling and comfort in sharing their experiences. One focus group of three participants (~2 h in length) and seven individual interviews (~1 h in length) were conducted using the University of Calgary Zoom platform and audio recorded, and notes were taken. Before the COLLECT focus groups or interviews, participants were asked to provide their consent to participate (orally or electronically based on their preference) and completed a demographic form hosted on the University of Calgary Qualtrics platform. The form was anonymous (and anonymised by the PaCER researchers) and not linked to the interview or focus group responses. All focus groups and individual interviews were conducted using REB‐approved semi‐structured question guides with open‐ended questions to allow for diverse responses and opportunities for probing questions (see Appendix 2 for the COLLECT focus group and interview guide). The PaCER researchers organised and conducted all focus groups and interviews.

#### 2.4.2 Thematic and Narrative Analysis

As part of the PaCER process, described by Marlett and Emes (2010) [11], data were collectively and iteratively analysed via thematic analysis. Thematic analysis is an approach developed by Braun and Clarke (2021) [12] that offers a flexible approach to identifying patterns and relationships within qualitative datasets. The PaCER researchers conducted a thematic analysis on all COLLECT data, including the focus group and all interviews. All transcripts were organised into an Excel file. After the initial focus group, all researchers coded the transcript using Braun and Clarke's methods to identify and consolidate initial codes and create a master codebook that could be applied to code further interviews. All subsequent interviews were coded by two PaCER researchers, and if any new codes were identified, they were added to the master codebook after being validated by two other PaCER researchers. Final codes were decided on between the pair of coders; in some cases, an extra coder was brought in if consensus could not be reached. After the coding was complete, all exemplar quotes that had been identified were also incorporated into a document organised by each code, so final counts could be made. At least one narrative analysis [11] was also conducted by each PaCER researcher on a whole interview or part of an interview to add additional context and background to the analysis. Through an iterative, interpretive process, all initial codes were refined and retained as sub‐themes, which were then collaboratively grouped under eight overarching themes by the PaCER researchers. Definitions and exemplar quotes for each theme and sub‐theme were chosen by consensus of the PaCER researchers. Once themes were finalised by the researchers, recommendations for policy and practice change that aligned with the major themes were created.

#### 2.4.3 REFLECT Phase

Individuals recruited to participate in the COLLECT focus group or interviews were also invited to voluntarily participate in the REFLECT focus group or interviews. This was to ensure that participants' experiences were accurately represented and then refined accordingly. After initial analysis, preliminary themes, sub‐themes and recommendations were shared in REFLECT focus groups and interviews (see Appendix 3 for the REFLECT focus group and interview guide). There was a total of five COLLECT study participants who also participated in REFLECT—three for a focus group and two for an individual interview. Together with the REFLECT participants, the PaCER researchers reached a common understanding of the findings and suggested future research directions, knowledge dissemination strategies and specific changes to the themes and recommendations. As a result, eight themes and twenty‐four sub‐themes, along with eight recommendations, were finalised based on the feedback.

## 3 Results

### 3.1 Participant Characteristics

The total sample size from the COLLECT phase was 10 participants (see Table 1). The sample was predominantly female (*n* = 7). Most participants were under the age of 23 (*n* = 8), with one participant aged 24–26 and one aged 31–35. The majority of participants resided in Alberta (*n* = 5) and Ontario (*n* = 4), with one participant from Saskatchewan. Additionally, most participants reported annual household income above $80,000 (*n* = 6), which we considered reflective of a higher economic background, with only one reporting an annual income of less than $20,000. Some participants self‐reported having more than one autoimmune condition (*n* = 2). There was some diversity in the types of autoimmune conditions, with the majority being gastrointestinal‐based conditions such as coeliac disease and inflammatory bowel disease (*n* = 6). Finally, all participants had attended university, and some had also attended graduate school within the last five years (*n* = 3).

**Table 1:** COLLECT participant (*n* = 10) demographics.

Characteristics	# of participants
Gender	
Male	3
Female	7
Age	
18–20	5
21–23	3
24–26	1
27–30	—
31–35	1
Current province of residence	
Alberta	5
Ontario	4
Saskatchewan	1
Annual household income	
Less than $20,000	1
$20,000–$79,999	2
$80,000 or more	6
Prefer not to answer	1
Chronic autoimmune conditions*	
Coeliac disease	3
Endometriosis	1
Inflammatory bowel disease	3
Juvenile idiopathic arthritis	2
Lupus	1
Secondary Sjögren's syndrome	1
Type 1 diabetes	1
Post‐secondary attendance or consideration	
Attended	10
Type of post‐secondary schooling attended or considered**	
University (undergraduate studies)	10
Graduate school	3

* Two participants self‐identified as having two chronic autoimmune conditions.

** All participants had attended or were currently attending university; three participants had also attended or were currently attending graduate school.

### 3.2 Themes and Sub‐Themes

Through initial analysis of patient‐participant experiences and feedback through the REFLECT stage, eight key themes were identified: ‘Protective/Risk Factors in Post‐Secondary Experience’, ‘Community Support’, ‘Information, Education, and Awareness of Autoimmune Conditions’, ‘Condition Impacts on Post‐Secondary Education Experience’, ‘Burden of Chronic Conditions’, ‘Recognising Student Resilience’, ‘Lessons of Covid‐19’, and ‘Direct Participant Feedback’ (see Table 2 for detailed definitions and exemplar quotes). During coding, no new codes were identified in the final interviews, suggesting that data and thematic saturation were achieved within these 10 participants; therefore, recruitment was stopped [13].

**Table 2:** Sub‐theme definitions and exemplar quotes.

Theme (# of quotes*)	Sub‐theme	Sub‐theme definition	Exemplar quote
Protective/Risk Factors in Post‐Secondary Experience (126)	Familiar/Trusted Healthcare	Identifying previous positive experiences and understanding of clinical/health‐related care as a protective factor when managing their condition and navigating post‐secondary education.	‘I've been lucky enough to find a good team that works around my schedule for me. I had surgery this summer and they wanted to do it in the fall, but they were kind enough to take into consideration that I value my time with school, so they scheduled me in the summer when I didn't have any classes.’—INT 3
	Experience Managing Chronic Conditions	Identifying the length of history with the condition and the success of past/current disease management as influential factors when navigating post‐secondary education.	‘I think I'm also blessed that it hasn't been as bad as other people. I know that for some people it's super super taxing and I think I caught mine early, but I've been dealing with it since I've been 14. So, at this point it's just kind of something that became a routine, an everyday sort of thing.’—INT 3
	Active versus Inactive Disease	Recognised influence of navigating post‐secondary education while managing active disease versus when disease is inactive/in remission.	‘It's possible I didn't have as much energy to prepare and study as much as I did, for example in my masters, where I had been on the diet for a while and still not feeling 100%, but certainly much better than I was leading up to the diagnosis, so it's likely that it impacted some grades and performance.’—INT 2
	Unfamiliar/Negative Healthcare Experience	Identifying negative experiences with new or past clinical/health‐related care as risk factors when navigating post‐secondary education.	‘I think there should be more clarity from pediatric into adult care, just exactly what that transition looks like, but also the reassurance that just because you're heading into adult care doesn't mean it's all going to be put on you.’—INT 3
	Proximity	Identifying the location/distance between various supports from post‐secondary school as a crucial factor when managing a chronic condition.	‘Living away from home just seemed really overwhelming because I didn't really know how I was supposed to do everything for myself when I kind of felt barely functional in a lot of ways.’—INT 4
Community Support (84)	Family and Friend Support	Identifying family, friends and significant others as positive influential supports when managing a chronic condition and navigating post‐secondary education.	‘I have friends who are not in the same program as me and I'm able to vent to them and they're always understanding and supportive.’—INT 6
	Peer Support	Identifying connection to fellow students, faculty, community members or mentors who share lived experience with similar conditions as a valuable protective factor and a desired support.	‘I think just seeing people in post‐secondary in a similar setting to what I was pursuing and then those that were later in their life and careers and still doing quite well, kind of gave some inspiration to keep going.’—INT 2
Information, Education and Awareness of Autoimmune Conditions (104)	Knowledge of Post‐Secondary School Resources/Supports	Identifying gaps in student awareness or understanding of resources and/or supports offered or provided by the post‐secondary school.	‘There's something that makes me feel kind of guilty about seeking help from accessibility services when I feel like there's people with actual learning disabilities or physical disabilities to, I don't know, be taking away resources from people like that.’—INT 4
	Benefits of Information and Awareness	Identifying the positive influence of available information resources for personal or societal use when managing and understanding chronic autoimmune conditions.	‘Of her own volition started researching it and read a lot about it from Red Cross, from the Canadian Celiac Association, so in that case literacy or literature from those kind of public outreach bodies is actually doing something.’—INT 4
	Lack of Education and Awareness	Identifying the negative influence of societal and post‐secondary misunderstanding or lack of knowledge surrounding chronic autoimmune conditions on students living with these conditions.	‘I feel for sure if there was more education about it, because I don't think it's talked about enough at all, then people could, it would be more normalised.’—INT 3
Condition Impacts on Post‐Secondary Experience (180)	Nutrition	Identifying nutrition, nutritional factors relating to disease management, and nutritional supports and/or resources as influential factors when navigating post‐secondary education.	‘Meal planning is something that takes a lot of time out of my day just because it's not convenient for me to eat anywhere so I have to make sure that I have food made for me.’—INT 3
	Financial Support	Identifying financial influence on student ability to manage chronic conditions while navigating post‐secondary education.	‘Having opportunities for people that just don't feel or aren't capable to do a full course load because of their health; missing out on those financial opportunities can be just another challenge that's added.’—INT 2
	Condition Impacting Mental Health	Identifying the influence that managing chronic conditions while navigating post‐secondary education has on student mental health.	‘It's definitely taken a toll on my mental health just because I see everybody living their life without these symptoms, and it really sucks to have to watch them go through the same schooling as me but have an easier time because they don't go through this like once or twice a week.’—INT 1
	Condition Impacting Academics/Academic Opportunities	Identifying the influence that managing chronic conditions while navigating post‐secondary education has on student academic attendance, performance and opportunities.	‘I'm kind of out for a week and feeling really strange and can't really think straight. I've definitely had to hand in assignments in that state and it just feels awful because I have no idea what this thing I'm producing is because I feel so weird and not myself.’—INT 4
	Condition Impacting Social Experience	Identifying the influence that managing chronic conditions while navigating post‐secondary education has on student social life.	‘I think the social aspect was one of the things that has impacted me the most, just because my friends will, we talked about eating or even just going out, and sometimes I can't because I actually get more tired than the average person.’—INT 3
Burden of Chronic Condition (27)	Challenges With Self‐Advocacy	Identified student difficulty in communicating the reality and needs of their condition to others.	‘Maybe it's partly because I would sort of minimise my condition in some ways. So I was like I don't think I can really go ask for maybe an extension on something or like can I do a test at a different time or like modify the assignment.’—INT 2
	Prioritising Post‐Secondary Experiences Over Health	Identifying circumstances requiring or felt to require primary attention of the student, while disease management was given lower importance.	‘I think just kind of letting sometimes physical health and mental health slip a little bit because there's so much more it seems more pressing and important to work on your courses or finish assignments.’—INT 2
Recognising Student Resilience (40)	Condition as Motivating Factor	Identifying aspects of living with chronic autoimmune conditions as influencing or inspiring positive drive when navigating post‐secondary education.	‘Originally my condition kind of maybe steered me away from doing something I was actually really passionate about. But now I kind of have managed it and I'm interested in helping other people. It's kind of provided motivation for my current post‐secondary path.’—INT 4
	Condition Not Primary Impact/Factor	Identifying the condition as non‐influential or other factors as having primary influence on the post‐secondary experience.	‘Crohn's disease for me is kind of something that doesn't really define me as a person, so I don't think it had too big of an impact on deciding whether I wanted to go to post‐secondary or not.’—INT 3
Lessons of Covid‐19 (12)	Impact of Covid‐19 (Negative)	The effects of Covid‐19 negatively impacted post‐secondary experiences.	‘It being online for half the year initially, it was tough to make more than superficial friendships … my support was instead of being from friends, was more from a resource seeking standpoint.’—FG1‐P1
	Impact of Covid‐19 (Positive)	The effects of Covid‐19 positively impacted post‐secondary experiences.	‘I started university during Covid, which was actually really helpful attendance wise, I think it was good when classes were online because you know you're at home so first of all there's no commute—it takes me around 40 min to get to the university so it was nice to not have to wake up super early and I felt more well rested and then the level of comfort I would have by being able to have my camera off and like move around or lie down and just make myself comfortable while I still studied.’—INT 6
Direct Participant Feedback (127)	Post‐Secondary School Not Meeting Needs	Identifying current supports and/or resources or lack of supports and/or resources offered by post‐secondary schools, as not meeting student needs when managing a chronic condition.	‘I think that my university would benefit from more robust supports for students with disabilities. There are accommodations out there, but they're actually a little bit difficult to find and a little bit difficult to apply for.’—INT 6
	Post‐Secondary School Resource/Support	Identifying helpful resources and/or supports accessed that were provided by post‐secondary schools.	‘And the school was super helpful. All they needed was the note from my doctor and I had a really great experience with the counsellor. She was very understanding along with my professors were very understanding. And none of them questioned my accommodations, which I think also really helped me. I was able to access the services without feeling more pressure or more stress, which would obviously just make my disease probably flare even worse.’—INT 7
	Participant Recommendations	Specific recommendations stated directly by research participants.	‘I think that on their part they should remove those barriers to access and then also increase the amount of outreach they do for students who are considering studying there or students who are early on in their degree.’—INT 6

* The number of quotes said by each participant per theme. If a quote fit under two themes, it was counted twice.

#### 3.2.1 Protective/Risk Factors in Post‐Secondary Experience

Positive or negative factors associated with chronic autoimmune conditions that greatly influence health outcomes and the experience of post‐secondary education were identified as a main theme. Familiar and trusted healthcare was a key protective factor, with positive care experiences helping students feel more supported in managing their chronic condition and post‐secondary education. Similarly, having their condition longer made it easier for students to handle academic challenges as they had more experience managing their symptoms. The difference between active versus inactive disease was also highlighted, as managing symptom flares presented more difficulties as compared to periods of remission. Conversely, negative or unfamiliar healthcare experiences acted as risk factors, adding stress. Proximity to supports was another important factor, as distance from usual supports, like family and friends, impacted students' ability to balance health and education. These protective and risk factors all have important implications for the success of students in post‐secondary education.

#### 3.2.2 Community Support

Another key theme was the importance of community and the sense of support it provides to students living with chronic autoimmune conditions. Family and friends were identified as crucial supports in managing their condition while navigating post‐secondary life.

‘*I did pick [university] specifically because I would stay here, I've never been keen on being away from my family, but it is because they're my biggest support system. I just feel like I'd be super lost and frazzled without them’* (INT‐3).

Additionally, peer support, particularly from those with similar lived experiences, was highly valued.

‘*Find a network of people that also have an autoimmune disease even if it's not the same one. Once I found support it was like truly life changing to know that other people were out there facing these same very niche experiences and problems’* (INT‐7).

These strong connections and supports were greatly valued among participants.

#### 3.2.3 Information, Education and Awareness of Autoimmune Conditions

This theme considers the overall understanding of chronic autoimmune conditions and the support and resources necessary for positive outcomes for the student living with the condition. This knowledge was highlighted as being necessary for all, including schools, healthcare professionals, family, peers and the students themselves. A gap in awareness of post‐secondary resources was noted, with some students not knowing if they were eligible for accessibility services due to the nature of their condition or simply not knowing how to even access these supports and resources. Study participants emphasised the benefits of accessible information, which improved their own understanding and that of peers, family, professors and others in their academic and personal environments. However, a lack of education and awareness in society and post‐secondary settings posed significant challenges for students managing these conditions.

‘*There was a professor who I asked for an extension … the professor had a remark about how now that my assignment is being handed in late, she has to mark it at a separate time … which I was very upset after she said that to me of course because I asked for an accommodation and now she's making it seem like I'm really burdening her*’ (INT‐6).

Overall, participants highlighted the need for effective information, education and awareness around chronic autoimmune conditions as well as supports and resources available.

#### 3.2.4 Condition Impacts on Post‐Secondary Experience

This theme describes the influence that chronic autoimmune conditions have on student success and outcomes when pursuing higher education. Many factors encompass this theme, including nutrition, where access to proper nutritional support and management was seen as critical. Finances were another key issue, with participants expressing concern about missing financial opportunities due to their condition. Mental health was frequently impacted by the stress of juggling disease management and academic demands. Participants also noted challenges in academic attendance and performance, often missing opportunities due to health‐related issues. Lastly, the impact on social experiences was significant, as many struggled to balance disease management with their social life. These factors were described as significantly shaping the post‐secondary experiences of participants, highlighting the need for greater attention to these challenges.

#### 3.2.5 Burden of Chronic Condition

This theme captures the personal cost and emotional labour associated with living with a chronic autoimmune condition while navigating post‐secondary education. Participants noted challenges with self‐advocacy, often minimising or avoiding disclosure of their condition to prevent being perceived as burdensome.

‘*New people that I meet, they don't really understand what that means. They don't know that I can't do as much as them. But that's my own fault, cause I don't really talk about it’* (INT‐3).

Additionally, some described situations where they felt compelled to prioritise their academic responsibilities over health, leading to further health strain, sometimes without fully recognising the trade‐off until later.

‘*It's almost like having a part‐time or even full‐time job going to appointments focusing on your medication, filling prescriptions, all of that so I was kind of worried about finding that balance and then I was worried that I would either prioritise my studies too much and then let my health fall to the wayside’* (INT‐6).

This underscores the internalised and often unexamined burden that many students living with chronic, invisible conditions carry. Recognising these obstacles allows for further understanding of the accommodations, resources and supports needed for success.

#### 3.2.6 Recognising Student Resilience

Acknowledging the resilience of students living with chronic autoimmune conditions while navigating higher education was highlighted as a powerful theme. Participants demonstrated remarkable perseverance, with some viewing their condition as a motivating factor, inspiring them to push forward.

‘*Growing up with arthritis definitely impacted what I wanted to study and especially knowing that I wanted to go into health care, knowing the care that I had received and being a patient for what was a lot of my life’* (INT‐7).

While others felt that their condition was not the primary influence on their post‐secondary experience, pointing to other personal, academic or social factors that shaped their educational path.

‘*Crohn's disease for me is kind of something that doesn't really define me as a person, so I don't think it had too big of an impact on deciding whether I wanted to go to post‐secondary or not’* (INT‐3).

This perspective highlights the determination and autonomy some students expressed, even without realising, choosing to focus on their goals and identities beyond their condition, despite the ongoing challenges they faced. This theme offers unique insights into the strength and adaptability of these students, highlighting resilience as a key element of their success.

#### 3.2.7 Lessons of Covid‐19

Although it is the least prominent theme, the pandemic posed a unique situation for all post‐secondary students, especially those with autoimmune conditions. This theme captures the negative and positive outcomes that resulted from the Covid‐19 pandemic for those with chronic autoimmune conditions attending university. Most notably, participants reflected on how certain accommodations they needed (e.g., virtual attendance options and recorded lectures) were now easily accessible during the pandemic.

‘*I was offered online because it was still in Covid. So, because of that, it was already going to be recorded, I already felt I had the resources that I need’* (INT‐7).

These reflections suggest that institutional adaptations made during the pandemic could serve as a model for more inclusive and sustainable accommodations moving forward.

#### 3.2.8 Direct Participant Feedback

This final theme includes any participant suggestions for new supports or resources and thoughts on existing post‐secondary supports or resources currently provided. Many participants felt that post‐secondary schools were not meeting their needs, highlighting gaps in available supports for managing their conditions. However, some did identify helpful resources provided by their schools that had positively impacted their academic experiences, which can be translated into recommendations for other post‐secondary schools. Importantly, participants also offered specific recommendations on how schools could better support students with chronic autoimmune conditions.

‘*We need resources where kids can connect with one another. I know at my school we don't have any support groups or anything like that where we can go together and be like I struggle with this…it needs to be normalised that you're not alone in these settings because it is very, very isolating’* (INT‐5).

This feedback serves as a powerful cross‐cutting thread, as many suggestions directly complement other themes, like community support (as demonstrated in the above quote), offering insight into how systemic issues might be addressed. Overall, this theme was instrumental in shaping participant‐informed recommendations (see Table 3), which also reflect insights gathered across multiple themes and were further refined through discussion in the REFLECT phase.

**Table 3:** Recommendations.

1. Cater supports and accommodations offered by the post‐secondary school to the individual student, involving family and healthcare providers whenever possible, and incorporate safety planning for various outcomes and shifts in the student's health and ability. This should involve identifying protective and risk factors of student success and planning to build or mitigate these, respectively.
2. Ensure that all messaging and marketing surrounding accommodations/accessibility services includes students living with chronic conditions specifically, and increase awareness of these services through orientations, post‐secondary website, online newsletters/emails, bulletin boards and so forth.
3. Increase information, education and awareness resources available on campus (including virtual) for students living with chronic conditions, their peers, their professors/instructors and other staff to develop skills, knowledge and understanding. This should include trainings, workshops, social gatherings, online newsletters/emails/blog posts, bulletin boards and so forth.
4. Ensure that various dietary food options are available on campus and at events, that information related to the preparation of foods is available, and that students can feel safe eating on campus by including third‐party monitoring or certification of dietary requirements.
5. Provide specialised mental health support options for students living with chronic conditions while attending post‐secondary school, such as trained, designated or specialised counsellors, peer counsellors, peer support options, and opportunities for students to build their capacity to enforce boundaries and advocate for their needs.
6. Ensure supports/accommodations recognise the fluctuating reality of living with chronic conditions and are flexible enough to meet these needs in terms of academic achievement and opportunities, including extensions, flexible schedule options and so forth. Continue to provide supports implemented due to Covid‐19 that were beneficial to students with chronic conditions and accessibility concerns.
7. Ensure post‐secondary social events are inclusive of and accessible to students with chronic conditions; gather feedback from the student body on areas to improve, including virtual event attendance, inclusive food options and so forth, and provide/host peer support opportunities through groups, clubs, mentors, safe spaces and virtual connections.
8. Recognise and encourage the strength and resilience of students when navigating post‐secondary with chronic conditions through awards, scholarships, project opportunities, shout‐outs, features in blogs or newsletters and so forth.

### 3.3 REFLECT Findings

From the REFLECT phase findings, it was evident that participants were in agreement on the final themes and sub‐themes. REFLECT participants suggested several directions for future research (see Section 6). Much of the discussion focused on considerations for change. Based on this feedback and all study participant experiences, eight specific recommendations were consolidated (see Table 3).

## 4 Discussion

Given the lack of understanding around chronic autoimmune conditions being an ‘invisible’ condition and the unique challenges and experiences this presents, this study aimed to address the experiences of young adults living with varying chronic autoimmune conditions transitioning into and throughout post‐secondary education. In relation to existing literature, this study offers a unique contribution. There is limited evidence on the topic of patient experiences with post‐secondary education while living with varying chronic autoimmune conditions, particularly in relation to current supports and resources. Therefore, these results are valuable in contributing to the evidence base in this important area of study.

Most identified themes and sub‐themes had support in previous qualitative studies [1, 2, 3, 14, 15] with other chronic conditions and disabilities. For example, Mullins and Preyde (2013) described the fluctuating nature of disability and how changes in health status can disrupt educational experiences [2]—a concept that closely parallels our themes of ‘Burden of Chronic Condition’, ‘Condition impacting academics/academic opportunities’ and ‘Active versus inactive disease’. Their work also highlighted the barriers associated with living with a disability that can lead to changes in mental health, such as social and organisational barriers [2], which echoes our themes surrounding conditions impacting social experience and mental health, and lack of education and awareness. Similarly, Hamilton et al. (2021) and Murray et al. (2019) identified that students with disability and chronic conditions are often not prioritised at post‐secondary schools, as there can be a lack of knowledge about how to best support impacted students [1, 3], which aligns with our sub‐theme highlighting lack of education and awareness. Hamilton et al. (2021) also described the positive perceptions of those living with chronic conditions [3]. This is reflected in our participants' experiences around resilience, specifically in our sub‐theme that discusses their chronic condition acting as a motivating factor, where participants expressed that living with their condition has inspired them to educate others about their lived experience and explore different career paths. Support from family and friends was another major theme shown throughout other studies [1, 14]. Notably, feelings of isolation were described in study participants who felt that they had a lack of support from family and friends [1], which aligns with our overall theme of community support, encompassing sub‐themes around family and friend support. Another key sub‐theme revealed throughout this study was challenges with self‐advocacy, where participants described difficulty communicating their needs to others. Allemang et al. (2020) also address this theme, emphasising the importance of self‐advocacy skill development on health outcomes and post‐secondary success [15]. Their study further identified the importance of knowledge of available services for those with chronic conditions and the positive outcomes this had on participants, such as feelings of support [15]. This directly supports our sub‐theme of knowledge of post‐secondary school supports and resources, which outlines how many participants had a gap in their awareness of relevant supports at their post‐secondary schools.

The increasing recognition of dyslexia in post‐secondary settings [7] offers a useful example of how institutional awareness, accommodations and support structures can evolve over time, highlighting the potential for similar progress to be made for students with chronic autoimmune conditions. Literature on accommodations for neurodiverse students offers practical strategies that may benefit students with chronic autoimmune conditions, such as extended deadlines, flexible attendance policies and peer support [16]—all ideas our participants highlighted as potentially being beneficial. This further demonstrates how schools can reduce barriers through structural change. Similarly, healthcare transition research emphasises the need for anticipatory guidance and skill‐building, such as helping youth practice self‐advocacy, understand their condition and navigate the health system [17], which are highly transferable skills that can also support success in post‐secondary education. This underscores the benefits of healthcare transition programmes and their potential to better prepare students with autoimmune conditions to navigate the post‐secondary transition. Studies on the transition into employment further highlight the importance of flexibility, remote work options and disclosure coaching to support young adults managing fluctuating health [18]. These examples parallel the recommendations raised by our participants and suggest that approaches from adjacent domains could be adapted to better meet the needs of students with chronic autoimmune conditions.

There are a few novel themes from this study that have not yet been described in other studies, particularly themes surrounding the Covid‐19 pandemic. Participants discussed the positive and negative impacts of Covid‐19 on their chronic autoimmune conditions and post‐secondary education experiences. Learning strategies and models developed during the Covid‐19 pandemic proved to be useful supports and tools for study participants, such as recorded lectures, and were promoted by participants to be continued. This is a relevant theme that arose from this study that is currently lacking in existing research.

The rich discussion within the focus groups and interviews helped participants explore how their chronic condition impacted their post‐secondary experience in ways they had not considered before. They acknowledged the all‐around lack of information, education and awareness for students living with chronic autoimmune conditions, from their peers, professors/instructors, accessibility advisers and even healthcare providers. Participants offered many important suggestions that would be beneficial if implemented into practice within the post‐secondary education system, such as: providing students with more awareness of resources available from the very start of their post‐secondary career; for example, healthcare providers that patients see regularly should have information to share about the various available post‐secondary supports. Professors and instructors, as well as post‐secondary schools, should adopt a principles‐based approach, grounded in awareness of invisible conditions, flexibility in accommodations and clear referral pathways to help ensure equitable support for students with chronic autoimmune conditions. Participants expressed that student accessibility services should be clear on supports available for students living with ‘invisible’ chronic conditions, as they often feel it's ‘not for them’ or does not accommodate their needs due to a lack of understanding. The term ‘chronic health condition’ may be important to use instead of or along with ‘chronic disability’, as participants expressed how they may not consider their diagnosis a disability. There is evidently a stigma surrounding the term disability, further emphasising the need for education and awareness surrounding invisible conditions, like autoimmune conditions. Students should be given the opportunity to provide feedback about their needs and whether they are being met. Specialised support available on campus for those living with chronic conditions, such as mental health or dietitian support, could be of great benefit. Having patient advocates and peer support to provide accessibility services with more knowledge and understanding around ‘invisible’ health conditions should be an important consideration. Many participants expressed that there should be a scholarship or funding opportunity for students living with chronic autoimmune conditions, recognising their strength and resilience. Having the option to study online, specifically recording lectures, provides students with the flexibility necessary to succeed while managing symptoms and should be considered. This study has demonstrated that ‘equity’ may not be demonstrated at all post‐secondary schools, especially as they relate to accessibility, as there is a lack of understanding and specific supports and resources for students living with chronic autoimmune conditions.

## 5 Strengths and Limitations

This study was strengthened with a diverse range of participants, particularly the recruitment of three male participants, as many patient‐centred qualitative research tends to have more success in recruiting those who identify as female, as seen in previous PaCER projects in similar population groups [19]. Despite recruitment efforts, no participants who had only considered and not attended post‐secondary education enrolled in the study. Additionally, only participants who attended university and graduate school participated, which contributed to gaps in perspectives within the research. In addition, chronic autoimmune conditions can manifest in diverse ways, and each individual tends to have their own unique experience with their condition. While all participants had lived with their condition for at least one year, we did not systematically collect data on true duration, which may have influenced participants' experiences. Due to a smaller sample size of participants, the incorporation of a wide array of experiences and conditions was limited, especially due to the high number of gastrointestinal‐based conditions. While not a primary aim of the study, thematic saturation appeared to have been reached, as no new codes or themes were identified during the final stages of analysis [13]. Although many of the challenges described may be transferable to other invisible chronic conditions, they should not be assumed to represent the experiences of all individuals with autoimmune conditions. While the goal of qualitative research is not to achieve representativeness, we recognise that our sample may not reflect the full diversity of young adults in Canada living with autoimmune conditions, particularly in terms of socio‐economic and educational background. While several common themes were shared across participants, we acknowledge that using a broad age range (18–35 years) may overlook potential age‐related differences in life stage, support needs or motivations for attending post‐secondary education. Struggles with recruitment also served as a limitation, especially in being able to run focus groups. This was due to finding times that worked for all young adults to attend, participant comfort in groups, and time zone differences between provinces. This restricted the research team to conducting many individual interviews, but this also allowed for a further richness of data and connection to each patient‐participant. This patient‐led research, conducted by the seven PaCER researchers, with diverse lived experiences, integrated multiple patient perspectives and individual health experiences throughout the entire research process (see Table 4 for GRIPP2‐SF). Despite the strengths of patient researchers, the fluctuating health due to the nature of living with a chronic autoimmune condition was a limitation.

**Table 4:** GRIPP2‐SF checklist for reporting patient and public involvement.

GRIPP2‐SF Item	Summary of patient and public involvement (PPI)
**Aim**—What was the aim of PPI in the study?	The aim of involving patients was to ensure the research reflected lived experiences of young adults with chronic conditions and to co‐develop a study that was relevant, meaningful and actionable for this population.
**Methods**—How were patients and the public involved in the study?	The study was entirely patient‐led; the seven PaCER researchers (patients trained in qualitative research) led the study's design, data collection, analysis and interpretation. Additionally, three external patient‐partners contributed to the refinement of the study design.
**Study Results**—How were patients and the public involved in the interpretation or dissemination of results?	In addition to the PaCER researchers leading the interpretation and dissemination of results, five study participants returned to assist in the refinement of themes and recommendations to validate findings. Their feedback directly informed the final recommendations and shaped the discussion.
**Discussion**—What was the impact of PPI on the study overall?	PPI helped define the scope of the study, ensuring it focused specifically on autoimmune conditions rather than a broader chronic condition category. The lived experiences of the research team enriched data interpretation and the relevance of final recommendations.
**Reflection and Limitations**—What went well and what could be improved in future PPI work?	The collaboration between patient‐partners and PaCER researchers added significant depth and authenticity to the study. The use of lived experience throughout all phases, from study design to analysis, was a major strength. However, future work would benefit from larger research teams, increased flexibility and tailored support for patient researchers managing unpredictable conditions such as autoimmune conditions. Providing adequate time and structural accommodations is key to ensuring inclusive and sustainable engagement, similar to post‐secondary settings.

## 6 Future Research

Looking ahead, several key areas for future research have been identified based on the findings of this study. This study aimed to collect data from a variety of chronic autoimmune conditions, limiting the ability to solely focus on the experiences of specific groups, for example, rheumatic diseases. Future research should be aimed towards specific autoimmune populations, rather than generalising this study to all autoimmune conditions, as each condition may have varying experiences and supports specific to their lived experience. In addition, future directions could be focused on participants pursuing different levels and types of post‐secondary education, as our study was limited to university students. This includes but is not limited to bachelor's, master's, PhD and post‐doctoral programmes as well as colleges, trade schools and so forth. Working across sectors and disciplines could make post‐secondary education for people living with chronic conditions more accessible, manageable and sustainable. Future research should implement cross‐sectoral programmes and assess the impact on people living with chronic conditions. Altogether, this data could be used to help post‐secondary schools implement enhanced and individualised accessibility services to improve post‐secondary experiences for those living with chronic autoimmune conditions.

## 7 Conclusion

The transition to post‐secondary education presents significant challenges for young adults with chronic autoimmune conditions, particularly in balancing health and academic‐related experiences. This study highlights a gap in existing research on invisible conditions, like autoimmune conditions, addressing the unique needs of these students in academic settings. Our findings indicate that many of their needs, especially around accessibility and support, are not being adequately met, affecting both academic success and overall well‐being. Future research should build on these findings to further explore how best to address these challenges, ultimately creating a more equitable and supportive environment for students with chronic autoimmune conditions in post‐secondary education.

## Author Contributions

**Samantha A. Morin:** Conceptualisation; Data curation; Formal analysis; Investigation; Methodology; Project administration; Resources; Validation; Visualisation; Writing – original draft; Writing – review & editing. **Angelina Horta:** Conceptualisation; Data curation; Formal analysis; Investigation; Methodology; Project administration; Resources; Validation; Visualisation; Writing – review & editing. **Katelyn Greer:** Conceptualisation; Data curation; Formal analysis; Investigation; Methodology; Project administration; Resources; Validation; Visualisation; Writing – review & editing. **Parveen Priya Rai:** Conceptualisation; Data curation; Formal analysis; Investigation; Methodology; Project administration; Resources; Validation; Visualisation; Writing – review & editing. **Haley Gross:** Conceptualisation; Data curation; Formal analysis; Investigation; Methodology; Project administration; Resources; Validation; Visualisation; Writing – review & editing. **Raegan Reiter:** Conceptualisation; Data curation; Formal analysis; Investigation; Methodology; Project administration; Resources; Validation; Visualisation; Writing – review & editing. **Ingrid Nielssen:** Conceptualisation; Methodology; Project administration; Resources; Supervision; Validation; Writing – review & editing. **Marcia Bruce:** Conceptualisation; Methodology; Project administration; Resources; Supervision; Validation; Writing – review & editing. **Kim Giroux:** Conceptualisation; Methodology; Project administration; Resources; Supervision; Validation; Writing – review & editing. **Deborah A. Marshall:** Conceptualisation; Methodology; Funding acquisition; Supervision; Resources; Project administration; Validation; Writing – review & editing.

## Ethics Statement

This study was approved by the University of Calgary Conjoint Health Research Ethics Board (REB23‐0906).

## Consent

All patient‐participants freely provided informed consent to participate in this study.

## Conflicts of Interest

Samantha A. Morin, Angelina Horta, Katelyn Greer, Parveen Priya Rai, Ingrid Nielssen, Haley Gross, Marcia Bruce and Kim Giroux have no conflicts of interest.

Deborah A. Marshall reports personal fees from the Office of Health Economics, Astellas and Novartis and meeting expenses from ISPOR and Illumina. Marshall is a member of the PaCER Advisory Committee and otherwise has no conflicts of interest directly related to this manuscript.

## Supporting information

HEX2024‐6881 Appendix UNBLINDED.

## Data Availability

The authors have nothing to report.
