# Supplementary material for: Seeing the Invisible Resiliency (STIR): Chronic Autoimmune Conditions and Post‐Secondary Education Experiences in Young Adulthood
Source: Health Expect. 2025 Jul 11;28(4):e70332. doi: 10.1111/hex.70332 (PMC12248230; doi:10.1111/hex.70332)
Supplement: Supplementary file 1 — HEX2024‐6881 Appendix UNBLINDED. [file HEX-28-e70332-s001.docx]

**Appendix 1: SET Discussion Group Guide**

How is the experience of young adults navigating post-secondary education impacted by living with chronic illness?

| **Approx. Time** | **Description** |
| --- | --- |
| 5 min | Discussion Kickoff   - Introductions - Land acknowledgement - Establish ground rules |
| 5 min | PaCER Process & Our Research Question   - PaCER methodology - Defining our research question - Research summary/background |
| 1 hour – 1 hour & 45 min | Discussion Questions  1. In one sentence – why were you interested in joining this discussion today?  2. Do you feel our research question will or will not create meaningful conversation? Do you feel anything is missing in our research question?  3. What do you think of our participant inclusion and exclusion criteria?  4. What do you think about our recruitment poster?  5. Do you have any suggestions of individuals or organizations we should share with for recruitment purposes?  6. Do you feel it would be helpful to provide our questions in advance of the focus group?  7. Do you have any other comments / advice for us? |
| 5 min | Final Wrap Up & Thank You |

**Appendix 2: COLLECT Focus Group & Interview Guide**

**Chronic Autoimmune Disease and Post-Secondary Education Experiences in Young Adulthood**

*Research Question: What are the experiences of young adults living with chronic autoimmune disease in accessing supports and resources for attending or considering attending post-secondary education?*

| **Approx. Time** | **Activity** |
| --- | --- |
| 20 minutes | Welcome, Introductions, Administrative   - Seeing the Invisible Resiliency (STIR) Team interviewers to introduce themselves and their role - Invite participants to introduce themselves - Complete any housekeeping tasks - Ask participants if there are any questions |
| 10 minutes | Purpose of this focus group:   - Describe the background and purpose of the focus group |
| 10 minutes | Summarize how we developed our research question   - Provide background of how the research question was developed and the Patient and Community Engagement Research (PaCER) process |
| 45 minutes | **Question Guide:**   1. How did your chronic auto-immune disease(s) impact or influence your decision when considering post-secondary education? 2. Can you describe your experience with looking for and getting resources/help for your chronic autoimmune disease(s) to support your post-secondary education?  - What kind of support did you receive? (Doctors, financial, friends, schooling, family, emotional, medical, community, educational) - Who was involved that made application or attendance easier or even possible (healthcare professionals, social workers, family, community, or other organizations)?  1. What were some challenges you had when attending or considering post-secondary education with your disease(s)? 2. What things helped you to explore post-secondary options? What things made you think this could be possible? 3. To what extent did your chronic auto-immune disease(s) impact your attendance and experience of post-secondary education? 4. What suggestions would you have to improve the experience of attending or considering post-secondary education while experiencing autoimmune disease(s)? |
| 15 minutes | Summarize the information collected |
| 20 minutes | Wrap up and discuss the engagement   - - - What will you take away from this session?     - Is there anything you would like to add?     - Is there anything we didn’t ask that you think we should have? |
| **TOTAL: 2hrs** |  |

**Appendix 3: REFLECT Focus Group & Interview Guide**

**Chronic Autoimmune Disease and Post-Secondary Education Experiences in Young Adulthood**

*Research Question: What are the experiences of young adults living with chronic autoimmune disease in accessing supports and resources for attending or considering attending post-secondary education?*

| **Time (will vary)** | **Activity** |
| --- | --- |
| 5 minutes | Welcome, Introductions, Administrative   - Thank patients for participating - Team introduction - Consent to record, demographic survey (if there are any new participants) - Remind participants they can withdraw |
| 5 minutes | Purpose of RELFECT Focus group   - The REFLECT focus group and interview create opportunities for patients participating in to come to an understanding of the findings and make suggestions on future research directions and knowledge dissemination - Confirm we captured key findings accurately, that we have not missed or misunderstood, and capture any key recommendations for going forward or ways we can best share our results - Remind the group that we are a safe space and are looking for honesty and respect in order to create a judgement-free zone. |
| 5 minutes | Summarize key findings from COLLECT |
| 35 minutes | **Question Guide:**  As a result of this work, we want to dive deeper to understand your thoughts on what this means and possible ways for us to share our findings.   1. What factors, outside of your chronic illness, may have also impacted your outlook and experience with post-secondary education? Is there anything we are missing that you would like added or changed? 2. What do the findings from the COLLECT focus group and interviews mean to you? 3. Is there anything you believe we were missing from our end? 4. What do you believe would be the best ways of sharing our findings to make an effective change in the system? Who should we share with? 5. To build on our research, do you have any ideas for future steps to take? |
| 10 minutes | Wrap up and validation of new discoveries through engagement:   - - - Tell us what you discovered today through this group conversation?     - If there is one key message that you hope we take away from our study, what would it be?     - Is there anything else you would like to further clarify or share?     - Thank participants for their time and engagement.     - Share any future goals with the group. |
